# Clinical value of resting cardiac dual-energy CT in patients suspected of coronary artery disease

**DOI:** 10.1186/s12880-022-00761-1

**Published:** 2022-02-27

**Authors:** Wenhuan Li, Fangfang Yu, Mingxi Liu, Chengxi Yan

**Affiliations:** 1 Department of Radiology, Beijing Chao-Yang Hospital, Capital Medical University, 8 Gongren Tiyuchang Nanlu, Chaoyang District, Beijing, 100020 China; 2 Department of Radiology, Xuanwu Hospital of Capital Medical University, No. 45, Chang-Chun Street, Xicheng District, Beijing, 100053 China

**Keywords:** Dual-energy CT, Myocardial perfusion, Coronary artery disease, Invasive coronary angiography, Positron emission tomography

## Abstract

**Background:**

Rest/stress myocardial CT perfusion (CTP) has high diagnostic value for coronary artery disease (CAD), but the additional value of resting CTP especially dual-energy CTP (DE-CTP) beyond coronary CT angiography (CCTA) in chest pain triage remains unclear. We aimed to evaluate the diagnostic accuracy of resting myocardial DE-CTP, and additional value in detecting CAD beyond CCTA (obstructive stenosis: ≥ 50%) in patients suspected of CAD.

**Methods:**

In this prespecified subanalysis of 54 patients, we included patients suspected of CAD referred to invasive coronary angiography (ICA). Diagnostic accuracy of resting myocardial DE-CTP in detecting myocardial perfusion defects was assessed using resting ^13^N-ammonia positron emission tomography (PET) as the gold standard. Diagnostic accuracy of cardiac dual-energy CT in detecting flow-limiting stenoses (justifying revascularization) by CCTA combined with resting myocardial DE-CTP, using ICA plus resting ^13^N-ammonia PET as the gold standard. The CCTA and DE-CTP datasets derived from a single-phase scan performed with dual-energy mode.

**Results:**

For detecting myocardial perfusion defects, DE-CTP demonstrated high diagnostic accuracy with a sensitivity, specificity, and area under the receiver operating characteristic curve (AUC) of 95.52%, 85.93%, and 0.907 on a per-segment basis. For detecting flow-limiting stenoses by CCTA alone, sensitivity, specificity, and AUC were 100%, 56.47%, and 0.777 respectively on a per-vessel basis. For detecting flow-limiting stenoses by CCTA combined with resting myocardial DE-CTP, sensitivity, specificity, and AUC were 96.10%, 95.29% and 0.956 respectively on a per-vessel basis. Additionally, CCTA combined with resting myocardial DE-CTP detected five patients (9%) with no obstructive stenosis but with myocardial perfusion defects confirmed by ICA plus ^13^N-ammonia PET.

**Conclusions:**

Resting cardiac DE-CTP demonstrates a high diagnostic accuracy in detecting myocardial perfusion defects and provides an additional clinical value by reducing rates of false-positive and false-negative patients beyond CCTA in patients suspected of CAD.

## Background

A comprehensive assessment of coronary artery disease (CAD) requires not only morphologic information about coronary artery stenosis but also functional evaluation about haemodynamic significance of coronary artery lesions [1–3]. Although a negative coronary CT angiography (CCTA) result provides excellent negative predictive value to exclude obstructive CAD (stenosis: ≥ 50%), the sole reliance on the presence of obstructive stenosis is less robust to identify CAD [4–6]. This can be caused by pathophysiologic explanations such as luminal thrombosis followed by recanalization, endothelial dysfunction with decreased coronary flow reserve, and vasospasm [7]. It can also be caused by technical factors such as inadequate resolution with heavily calcified plaque, branch vessel disease and image degradation during arrhythmia [8]. In any of these situations, myocardial perfusion assessment can provide complementary functional information in improving detecting CAD in patients presenting with chest pain.

Recent single-center studies have shown good diagnostic performance of rest/stress myocardial CT perfusion (CTP) in detecting myocardial ischemia and infarct in both the acute and stable outpatient groups [9]. But the additional value of resting CTP especially dual-energy CTP (DE-CTP) beyond CCTA in chest pain triage remains unclear. Therefore, the aim of our study was to evaluate the diagnostic accuracy of resting myocardial DE-CTP, and additional value in detecting CAD beyond CCTA in patients suspected of CAD presenting with chest pain.

## Methods

Our study was approved by the institutional ethics committee, and the written informed consent was obtained from each patient. The study design was retrospective. Patients were recruited from an existing prospective study database consisting of consecutive patients suspected of CAD presenting with acute or stable chest pain to our hospital between March 2017 and November 2019. Patients who underwent cardiac dual-energy CT scan, resting ^13^N-ammonia positron emission tomography (PET) and invasive coronary angiography (ICA) within one week time interval between examinations were enrolled. Patients were excluded if they had history of myocardial infarction, cardiomyopathy, myocarditis, contraindication to iodinated contrast agent, atrial fibrillation or renal dysfunction (estimated glomerular filtration rate < 60 ml/min/1.73 m^2^).

### PET data acquisition and image analyses

PET examinations were performed at rest on ECAT EXACT (CTI-Siemens, Knoxville, Tennessee, USA), which provide 47 tomographic slices. Patients abstained from food at least 6 h before the PET examination. Resting PET myocardial blood flow imaging was acquired 15 min after ^13^N-ammonia (555 MBq) was injected. Tomographic images were reconstructed by the filtered back projection method. Typical horizontal long axis, vertical long axis, and short axis tomographic views of the left ventricle were obtained by an image processing workstation for image analysis.

PET images were visually analyzed by 2 experienced readers who were blinded to the patient information. Horizontal long axis, vertical long axis, and short axis images were assessed on a per-segment and -territory basis using American Heart Association 17-segment model [10]. The disagreement of diagnosis between 2 readers was settled by a consensus reading. The disagreement of diagnosis between 2 readers was settled by a consensus reading.

### Invasive coronary angiography

ICA was performed by standard catheterization in accordance with the American College of Cardiology Guidelines for Coronary Angiography [11]. ICA was evaluated by quantitative coronary angiography (QCA; QuantCor QCA, Siemens AG Healthcare) by 2 cardiologists in consensus who were blinded to the dual-energy CT and PET results. All coronary artery stenosis was graded at least 2 orthogonal views and measurement was performed in the projection that showed the highest degree of stenosis. A mismatch between PET and ICA was defined as a positive PET scan with a negative ICA for significant coronary stenoses or a negative PET scan with a positive ICA for significant coronary stenoses.

### Dual-energy CT scan protocol

All CT examinations were performed at rest state using dual-energy mode of a 128-slice dual-source CT (DSCT; SOMATOM Definition Flash, Siemens Healthcare, Forchheim, Germany). Before the examination, the heart rate of each patient was measured. If the resting heart rate was higher than 65 beats per minute (bpm) and no contraindication to the use of β-blockers, metoprolol tartrate (Beloc, AstraZeneca, Wedel, Germany) was administered intravenously in fractions of 5–25 mg before the examination. Scanning parameters were as follows: 2 × 64 × 0.6 mm acquisition collimation with z-flying focal spot technique, and heart rate adaptive pitch of 0.17–0.35. Automated tube current modulation (Care Dose 4D, Siemens Healthcare) was used. One tube of DSCT system was operated with 165 reference mAs per rotation at 100 kV, and the second tube was automatically operated with 140 reference mAs per rotation at 140 kV. All scans were performed in cranio-caudal direction of supine position during a mid-inspiratory breath-hold.

The scanning range started from above the origin of the coronary arteries to below the dome of the diaphragm. Contrast agent was injected by a dual-syringe injector (Stellant D, Medrad, Indianola, USA) using an 18-gauge intravenous needle placed in the right antecubital vein. A triphasic injection protocol was used [12]. First, 50 mL of pure contrast media (Iopromide, Ultravist 370, 370 mg/mL, Bayer-Schering Pharma, Berlin, Germany) was administered. Thereafter, 30 mL of 70%/30% saline/contrast medium mixture was administered. Finally, 30 mL of saline was administered. The injection rate for all phases was 5 mL/s. Contrast agent application was controlled by a bolus tracking technique. A region of interest was placed in the aortic root, and image acquisition automatically started 7 s after the signal attenuation reached the predefined threshold of 100 Hounsfield units (HU).

### Dual-energy CT post-processing

CCTA and resting myocardial DE-CTP were reconstructed from data of single arterial phase dual-energy CT scanning. The CCTA images were reconstructed with 0.75 mm slice thickness, 0.5 mm increment, 75 ms temporal resolution, and B26f kernel. All images were reconstructed by the same person to reduce bias. Then the reconstructed CCTA images were transferred to multi-modality work-place (MMWP, Siemens Healthcare, Forchheim, Germany) and loaded into the Circulation application for further analysis.

The DE-CTP images were reconstructed with 1.5 mm slice thickness, 1.0 mm increment, 280 ms temporal resolution, and a dedicated dual-energy convolution kernel (D30f). By default, raw data were automatically reconstructed into low-kilovoltage (100 kV) images and high-kilovoltage (140 kV) images. Then the 100 kV and 140 kV images were transferred to MMWP and loaded into dual-energy Heart PBV (Siemens Healthcare) to calculate the iodine distribution maps, with color-coded of “Hot Body 8 bit”. A normal myocardial area was chosen to normalize the iodine distribution maps [13].

### Dual-energy CT image analyses

All dual-energy CT images were assessed on MMWP and patient information was removed. Images were assessed independently by two cardiac radiologists who were blinded to the patient information and disagreements were resolved by consensus. We performed separate readings of resting DE-CTP and CCTA. Readings were spaced at 2-week intervals to minimize recall bias.

The DE-CTP images were visually analyzed on a per-segment -territory and -patient basis according to the American Heart Association 17-segment model [10]. In color-coded iodine distribution maps, light orange indicated the highest iodine content and gray indicated the absence of iodine. For DE-CTP, the normal myocardium was defined as homogeneous light orange without any gray area; myocardial perfusion defect was defined as distinct grey area compared with normal surrounding myocardium. Diagnostic accuracy of resting myocardial DE-CTP in detecting myocardial perfusion defects was assessed using resting ^13^N-ammonia PET as the gold standard.

**Fig. 1:**
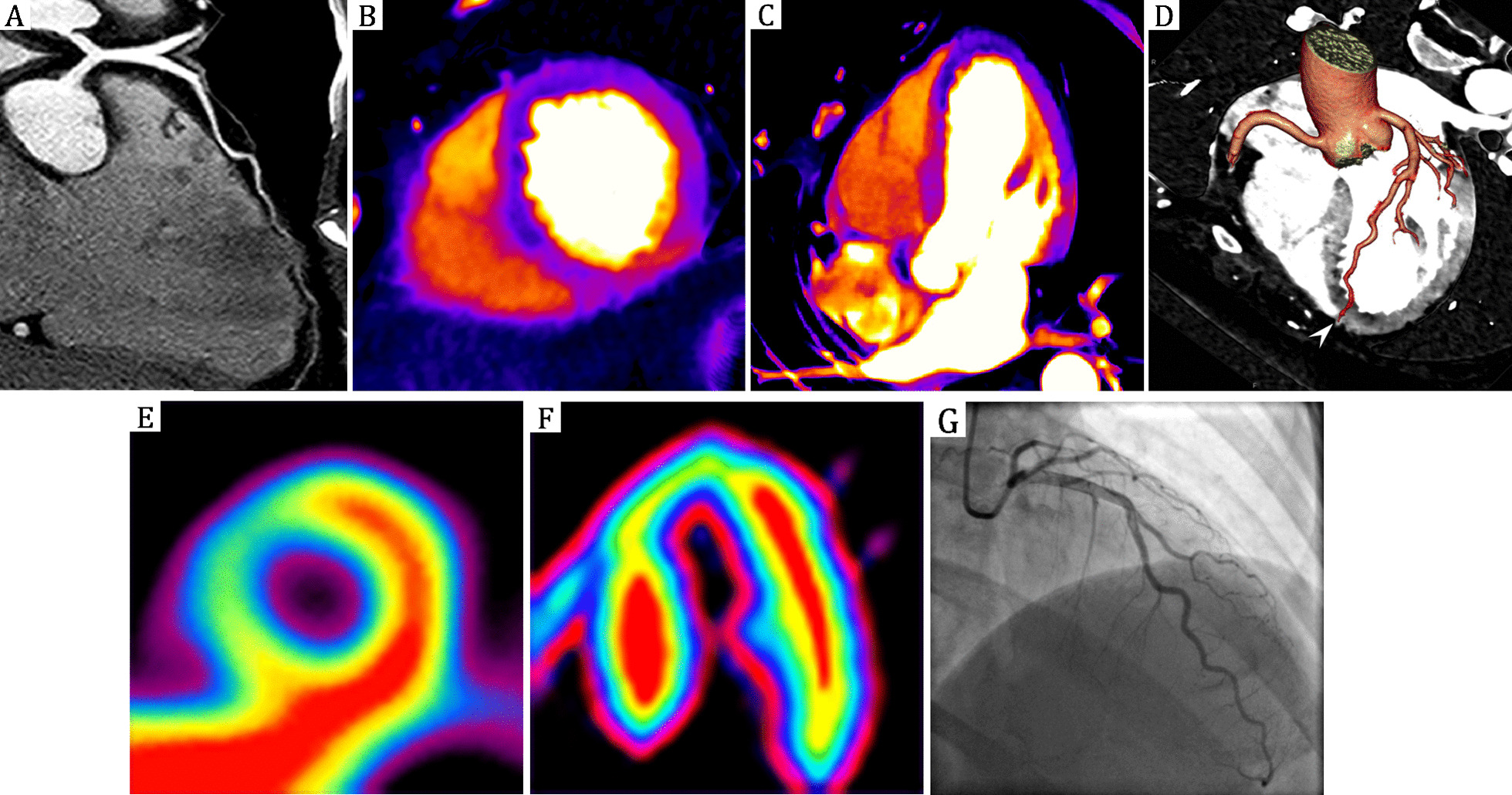
Mismatch between coronary stenosis severity and resting DE-CTP. **A** Curved multiplanar reconstruction CCTA reveals no coronary artery stenosis along left anterior descending (LAD). But resting DE-CTP analysis multiplanar reformatting demonstrates a rest perfusion defect in short-axis (**B**) and horizontal long-axis views (**C**), which confirmed by ^13^N-ammonia PET (**E, F**). In this 48-year-old female presenting with chest pain, serial troponin was mildly elevated leading to invasive coronary angiography (**G**), which revealed mild narrowing of the left anterior descending that resolved with intracoronary nitroglycerin, consistent with coronary vasospasm. **D** Fusion image of three-dimensional CCTA and two-dimensional DE-CTP shows the relationship between the perfusion defects area and corresponding supplying artery (LAD). DE-CTP = dual-energy CT perfusion; CCTA = coronary CT angiography; PET = positron emission tomography; LAD = left anterior artery

CCTA images were analyzed for coronary obstructive stenosis ≥ 50% for each vessel (left anterior descending coronary artery, right coronary artery, and left circumflex coronary artery) based on axial source images, cross-sectional views, multiplanar reformations, curved planar reformations and thin-slab maximum intensity projection images. The association between coronary artery distribution and myocardial segments was analyzed on the basis of the AHA recommendations [10]. In view of the possibility of the mismatch between vessels and segments based on the AHA model, curved planar reconstruction or three-dimensional CT renderings was used to establish one to one relationship between each myocardial segment and the coronary artery that supplies it, providing accurate registration between them (Fig. 1D). Diagnostic accuracy of cardiac dual-energy CT in detecting flow-limiting stenoses was assessed by CCTA combined with resting myocardial DE-CTP, using ICA plus resting ^13^N-ammonia PET as the gold standard.

### Statistical analysis

Statistical analysis was performed using SAS version 9.1 (SAS Institute Inc., Cary, North Carolina), and the threshold of significance was *P* value < 0.05. Quantitative variables were expressed as mean value ± standard deviation. The diagnostic performance was calculated, including sensitivity, specificity, positive predictive value, negative predictive value, and accuracy with 95% confidence intervals. Additionally, the receiver operating characteristic (ROC) curve analysis was performed. The area under the ROC curves (AUCs) were compared by the DeLong method. Kappa tests were used to assess intra- and interobserver agreement in CCTA and resting myocardial DE-CTP analysis in 10 randomly selected patients. The Kappa value was interpreted as follows: 0–0.20 poor agreement, 0.21–0.40 fair agreement, 0.41–0.60 moderate agreement, 0.61–0.8 good agreement, and > 0.81 excellent agreement.

## Results

**Fig. 2:**
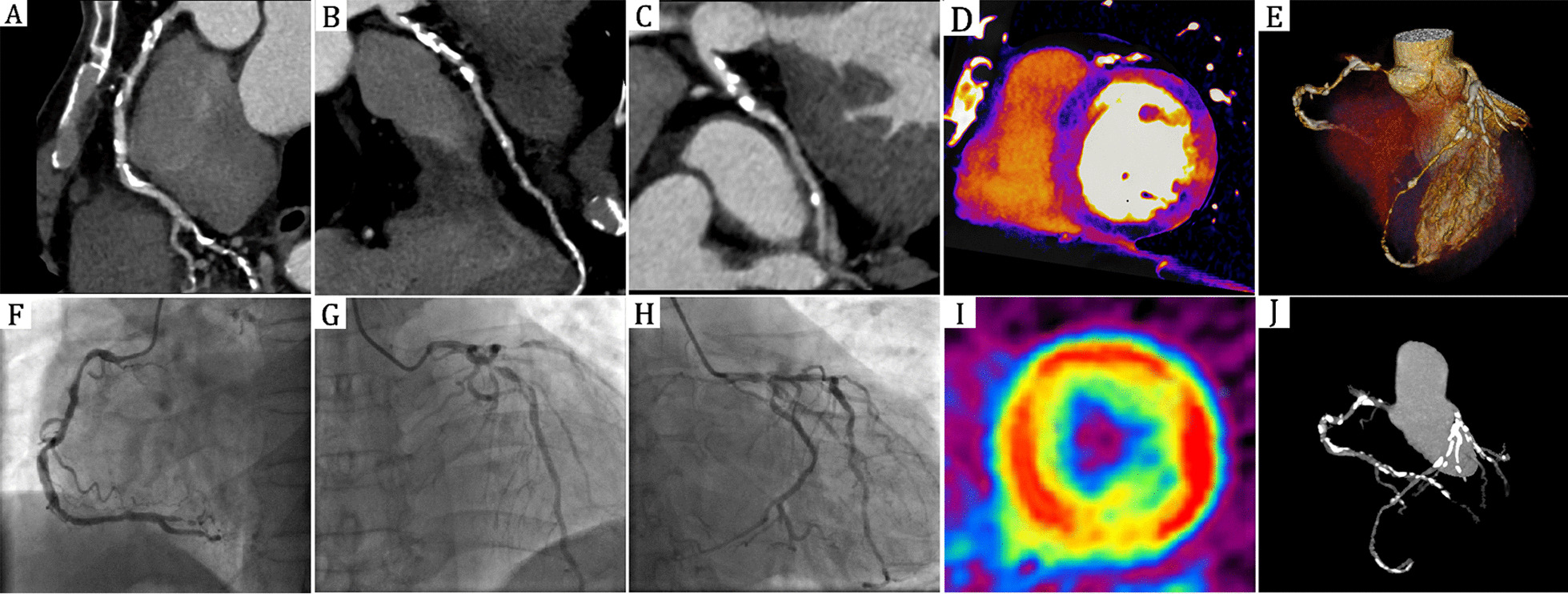
Match between coronary stenosis severity and resting DE-CTP. Curved multiplanar reconstruction CCTA reveals obstructive coronary artery disease with severe stenosis in RCA (**A**), LAD (**B**) and LCX (**C**). **D** Resting DE-CTP shows myocardial perfusion defects in anteroseptal, anterolateral, inferior walls and a subendocardial perfusion defect in inferolateral wall. This 63-year-old male subsequently underwent invasive coronary angiography (**F–H**) and PET (**I**) which confirmed the DE CT results. **E**, **J** are three-dimensional volume rendering technique (3D-VRT) and three-dimensional maximum intensity projection (3D-MIP) of CCTA respectively. DE-CTP = dual-energy CT perfusion; CCTA = coronary CT angiography; RCA = right coronary artery; LAD = left anterior artery, LCX = left anterior descending; PET = positron emission tomography

The characteristics of the study analytic population are summarized in Table 1 (n = 54). The radiation effective dose was 2.7 ± 0.5 mSv (dose-length product × 0.014 mSv/mGy·cm) for of dual-energy CT scanning. For the cardiac PET examination, the radiation dose from 15 mCi (555 MBq) of ^13^N-ammonia is 1.11 mSv. Figure 1 showed an example of mismatch between coronary stenosis severity and resting DE-CTP. Figure 2 showed an example of match between coronary stenosis severity and resting DE-CTP.

**Table 1:** Characteristics of the study population (n = 54 patients)

Characteristics	Value
Age (years) [mean ± SD; range]	60 ± 10 [39,76]
Sex [male/female]	32/22
BMI [kg/m^2^; mean ± SD; range]	25 ± 4 [21,30]
Mean heart rate during DECT (bpm) [mean ± SD; range]	59 ± 9 [45,78]
Hypertension (%)	18(33%)
Hypercholesterolemia (%)	12(22%)
Diabetes mellitus (%)	12(22%)
Current or prior cigarette smoking (%)	14(26%)

Values are n (%)

SD, standard deviations; BMI, body mass index; bpm, beats per minute

### Myocardial dual-energy CT perfusion in detecting myocardial perfusion defects

A total number of 918 myocardial segments in 162 territories in 54 patients were analyzed. Diagnostic accuracy, sensitivity, specificity, positive predictive value (PPV), negative predictive value (NPV), and AUC of DE-CTP in detecting myocardial perfusion defects were 89.43%, 95.52%, 85.93%, 79.60%, 97.09%, and 0.907 respectively on per-segment basis; 91.98%, 97.56%, 86.25%, 87.91%, 97.18%, and 0.919 respectively on a per-territory basis; 94.44%, 100%, 82.35%, 92.50%, 100%, and 0.912 respectively on a per-patient basis (Table 2).

**Table 2:** Diagnostic performance of resting DE-CTP

	Per segment (n = 918)	Per territory (n = 162)	Per patient (n = 54)
Accuracy	89.43 (821/918) [87.26–91.35]	91.98 (149/162) [86.67–95.66]	94.44 (51/54) [84.61–98.84]
Sensitivity	95.52 (320/335) [92.72–97.47]	97.56 (80/82) [91.47–99.70]	100.00 (37/37) [90.51–100.00]
Specificity	85.93 (501/583) [82.84–88.65]	86.25 (69/80) [76.73–92.93]	82.35 (14/17) [56.57–96.20]
PPV	79.60 (320/402) [75.32–83.44]	87.91 (80/91) [79.40–93.81]	92.50 (37/40) [79.61–98.43]
NPV	97.09 (501/516) [95.25–98.36]	97.18 (69/71) [90.19–99.66]	100 (14/14) [76.80–100]
AUC	0.907 [0.887–0.925]	0.919 [0.866–0.956]	0.912 [0.803–0.972]

Data are % (raw data) [95% confidence interval] for accuracy, sensitivity, specificity, PPV, and NPV. Data are value [95% confidence interval] for AUC

DE-CTP = dual-energy CT perfusion; PPV = positive predictive value; NPV = negative predictive value; AUC = area under the receiver operating characteristic curve

### Cardiac dual-energy CT in detecting flow-limiting coronary artery disease

**Fig. 3:**
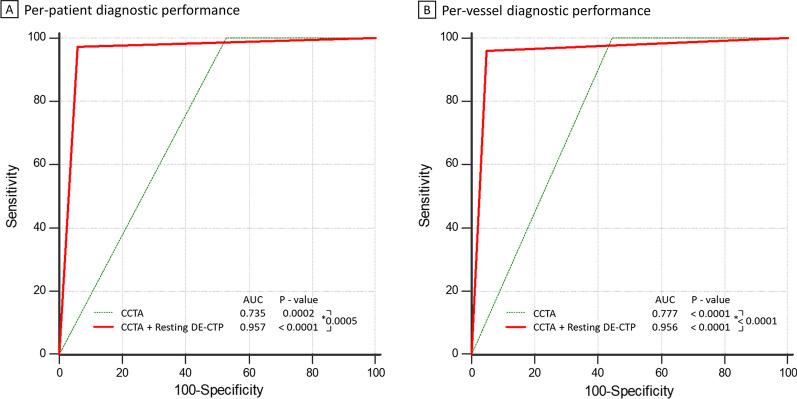
Area under the receiver operating characteristic curve (AUC) of per-patient (**A**) and per-vessel (**B**) performance of CCTA and CCTA plus resting DE-CTP for the detection of flow-limiting stenoses. ^★^P < 0.05 for comparison of AUC between CCTA and CCTA plus resting DE-CTP

On a per-patient basis, diagnostic accuracy, sensitivity, specificity, PPV, NPV and AUC in detecting flow-limiting stenoses were 83.33%, 100%, 47.06%, 80.43%, 100%, and 0.735 respectively for CCTA along; 96.30%, 97.30%, 94.12%, 97.30%, 94.12%, and 0.957 respectively for CCTA combined with resting myocardial DE-CTP (Table 3). On a per-vessel basis, diagnostic accuracy, sensitivity, specificity, PPV, NPV and AUC in detecting flow-limiting stenoses were 76.16%, 100%, 56.47%, 67.54%, 100%, and 0.777 respectively for CCTA along; 95.68%, 96.10%, 95.29%, 94.87%, 96.43%, and 0.956 respectively for CCTA combined with resting myocardial DE-CTP (Table 4). CCTA plus resting DE-CTP showed significantly better diagnostic performance than CCTA alone in the detection of flow-limiting stenoses on both a per-patient basis (AUC, 0.957 vs. 0.735, p = 0.0005) and per-vessel basis (AUC, 0.956 vs. 0.777, p < 0.0001) (Fig. 3).

**Table 3:** Per-patient diagnostic performance of CCTA and CCTA plus resting DE-CTP for detection of flow-limiting stenoses

	CCTA	CCTA plus resting DE-CTP
Accuracy	83.33 (45/54) [70.71–92.08]	96.30 (52/54) [87.25–99.55]
Sensitivity	100.00 (37/37) [90.51–100.00]	97.30 (36/37) [85.84–99.93]
Specificity	47.06 (8/17) [22.98–72.19]	94.12 (16/17) [71.31–99.85]
PPV	80.43 (37/46) [66.09–90.64]	97.30 (36/37) [85.84–99.93]
NPV	100 .00 (8/8) [63.06–100.00]	94.12 (16/17) [70.28–99.88]
AUC	0.735 [0.598–0.846]	0.957 [0.864–0.939]

Data are % (raw data) [95% confidence interval] for accuracy, sensitivity, specificity, PPV, and NPV; Data are value [95% confidence interval] for AUC

CCTA = coronary CT angiography; DE-CTP = dual-energy CT perfusion; PPV = positive predictive value; NPV = negative predictive value; AUC = area under the receiver operating characteristic curve

**Table 4:** Per-vessel diagnostic performance of CCTA and CCTA plus resting DE-CTP for detection of flow-limiting stenoses

	CCTA	CCTA plus resting DE-CTP
Accuracy	76.16 (125/162) [69.92–83.38]	95.68 (155/162) [89.72–97.43]
Sensitivity	100.00 (77/77) [95.32–100.00]	96.10 (74/77) [89.03–99.19]
Specificity	56.47 (48/85) [45.28–67.20]	95.29 (81/85) [88.39–98.70]
PPV	67.54 (77/114) [58.14–76.01]	94.87 (74/78) [87.39–98.59]
NPV	100.00 (48/48) [92.60–100.00]	96.43 (81/84) [89.92–99.26]
AUC	0.777 [0.705–0.839]	0.956 [0.912–0.982]

Data are % (raw data) [95% confidence interval] for accuracy, sensitivity, specificity, PPV, and NPV. Data are value [95% confidence interval] for AUC

CCTA = coronary CT angiography; DE-CTP = dual-energy CT perfusion; PPV = positive predictive value; NPV = negative predictive value; AUC = area under the receiver operating characteristic curve

When DE-CTP and CCTA were combined, the number of false positive patients with flow-limiting stenoses was reduced from nine to one, the PPV improved to 97.30% from 80.43%, and the specificity improved to 94.12% from 47.06%. Additionally, 5 in 54 (9%) patients showed myocardial perfusion defects with no obstructive stenosis on CCTA combined with DE-CTP and were confirmed by ICA combined with ^13^N-ammonia PET. That is to say that compared with CCTA along, DE-CTP combined with CCTA successfully avoided five false negative patients in our study.

The inter-observer agreement was “excellent” for DE-CTP (*k* = 0.90, *P* < 0.001) and on a per-territory basis, and was "excellent" for CCTA (*k* = 0.91, *P* < 0.001) on a per-vessel basis.

## Discussion

Our study demonstrates that resting cardiac DE-CTP has a high diagnostic accuracy in detecting myocardial perfusion defects and provides an additional clinical value by reducing rates of false-positive and false-negative patients beyond CCTA in patients suspected of CAD.

Our results are similar with previous studies in demonstrating an excellent ability for combined CTA and resting CTP in detecting flow-limiting coronary stenoses [8, 14]. Our results support the concept that resting DE-CTP provides additional value to include or exclude CAD beyond anatomic CCTA data. In comparison with their studies enrolled patients presenting with acute chest pain in emergency department, we enrolled patients presenting with acute or stable chest pain highly suspected of CAD and therefore had an expansion of different groups in evaluating the resting CTP in triage patients for invasive examination and treatment. Additionally, the CTP image set in their studies was from conventional CCTA datasets. Whereas, CTP in our study was the iodine maps from dual-energy scan mode. A previous work by our groups demonstrated that dual-energy CT iodine maps is superior to conventional CTP in detecting myocardial perfusion defects [15]. For DE-CTP, dual-energy CT iodine maps has great power to reduce beam-hardening artifacts and enhance difference between perfusion defects and health myocardium [9, 16, 17]. The current result also extends our previous study by combined CCTA with resting DE-CTP in detecting flow-limiting coronary stenoses in cohorts suspected CAD patients presenting with chest pain [15]. And above all, three-dimensional CT rendering used in current study established one to one relationship between each myocardial segment and the coronary artery that supplies it, providing accurate registration between them (Fig. 1D).

In keeping with prior reports, our results also demonstrate that in addition to improving ability of detecting flow-limiting coronary stenoses, combined CCTA with DE-CTP successfully detected five patients (9%) which abnormal perfusion without obstructive stenosis (≥ 50%) [18]. That is to say that 9% (5/54) patients of our study population refrained from false negative results. However, the prevalence of abnormal perfusion without obstructive stenosis was relatively lower than previous reports [19]. This may be because the prevalence abnormal perfusion without obstructive stenosis was more often in females than in males [20], whereas the percent of the female population (41%) was less than males in our study.

The diagnostic work-up of patients suspected of CAD at presentation is typically oriented toward the detection of a hemodynamically relevant obstructive stenosis, which serves as the basis for further treatment decisions. However, a considerable number of patients who have angina and signs of ischemia at presentation do not have significant obstructive disease, obstructive stenosis as defined by 50% or greater diameter stenosis [21–24]. Additionally, recurrent angina after successful percutaneous coronary intervention remains a clinically relevant issue occurring in a non-negligible percentage of patients [25].

Moreover, DE-CTP derived iodine maps allow a quantitative analysis of myocardial iodine uptake in mg/ mL which is directly proportional to myocardial blood supply that are additive to routine single-energy CT, which mostly provides density-based information [26, 27]. Future investigations should also evaluate their potential use in assessing and quantifying efficacy of coronary revascularization.

### Study limitations

Several limitations of this study should be described here. First, an absolute quantitative approach was not used to determine the myocardial perfusion defects. Second, the relatively small number of patients included in this study limits the statistical power and strength of the conclusions. This reflects the logistical difficulties involved in carrying out multimodality imaging. Although small, the results are encouraging but need to be tested on a larger cohort. Third, in view of technical intermodality differences between dual-energy CT and PET in the higher spatial resolution (namely, dual-energy CT), leading to slight differences in the visualisation of the transmurality of perfusion defects, we did non evaluate the transmurality of the perfusion defects. Fourth, ICA + PET is considered the gold standard in this study. Nevertheless, it should be acknowledged that ICA plus invasive fractional flow reserve (iFFR) is the unequivocal new gold standard for flow-limiting lesions.

## Conclusions

In this study, resting cardiac DE-CTP demonstrates a high diagnostic accuracy in detecting myocardial perfusion defects and provides an additional clinical value by reducing rates of false-positive and false-negative patients beyond CCTA in patients suspected of CAD. Future studies involving larger numbers of patients will be necessary for the validation of our findings.

## Data Availability

The datasets used and/or analysed during the current study are available from the corresponding author on reasonable request.
